# The accuracy of estimating chronological age from Demirjian and Nolla methods in a Portuguese and Spanish sample

**DOI:** 10.1186/1472-6831-14-160

**Published:** 2014-12-23

**Authors:** Luís F Tomás, Lisete SM Mónico, Inmaculada Tomás, Purificación Varela-Patiño, Benjamín Martin-Biedma

**Affiliations:** School of Medicine and Dentistry, University of Santiago de Compostela, Santiago de Compostela, Spain; Faculty of Psychology and Education Sciences, University of Coimbra, Coimbra, Portugal

**Keywords:** Nolla method, Demirjian method, Age estimation, Chronological age

## Abstract

**Background:**

Age determination has great importance in many clinical decisions, being commonly used in odontopediatrics, orthodontics, pediatrics, and forensic medicine. The Nolla and Demirjian et al. methods have been used for these purposes. However, estimating chronological age by means of the dental mineralization stage is not a straightforward analysis, and it is fundamental to test the validity of these methods and their applicability to populations. In this article we intend to compare the accuracy of estimating chronological age from dental age measured with the Nolla and Demirjian methods in a Portuguese and Spanish sample, considering the variables of sex and age-group.

**Methods:**

The sample was composed of 821 orthopantomographs of healthy Portuguese (n = 270) and Spanish (n = 551) subjects from 4 to 34 years old. For the Nolla and Demirjian methods, seven mandibular left teeth were examined, staged according to the dental maturity scale of each method. We obtained a good index of inter-rater agreement, a good internal consistency for the teeth assessment, and a good temporal consistency.

**Results:**

Dental age was calculated for each method. The Demirjian et al. method tends to overestimate the real age of participants and the Nolla method tends to underestimate it. The accuracy of both methods varied between the sexes and age groups. Both methods were found to be more precise with males. As the age-group increases, the predictive capacities of both methods diminish. The Nolla method was more accurate than the Demirjian method in early and late childhood for both sexes. Neither method could predict chronological age in adults.

**Conclusions:**

We can estimate chronological age for early and late childhood, through the Nolla and Demirjian methods, with the former showing greater predictive capacities than the latter. The Demirjian method tends to overestimate age and the Nolla method tends to underestimate it, leading to the importance of forming regression equations adapted to the population studied. Nolla and Demirjian formulas adapted to our sample were created as a function of sex and age-group.

## Background

Age determination has great importance in many clinical decisions, being commonly used in pediatrics, legal medicine, forensic sciences, anthropology, odontopediatrics and orthodontics [1, 2, 12, 13, 15, 16, 29, 31, 41, 42]. Increased immigration and mixing of populations, due to the globalized economy resulting from the increased migratory flow, sets legal problems of various orders, with increasing importance of determining the chronological age of children, youths and young adults. So estimating chronological age is important in assessing the legal adult age of people without documents for judicial purposes, in determining growth and somatic development, in orthodontic treatment and in the area of anthropology to estimate the age of past populations from immature skeletal remains [1, 2, 5, 6, 12, 13, 15, 16, 29, 31, 49].

Dental and bone age have been assessed to determine to what extent they are correlated for diagnostic purposes. Considering that states of dental mineralization are much less affected by environmental [2, 11, 46] and hormonal [2, 23, 28] variations than states of bone mineralization, dental development provides more reliable indications of chronological age than bone development [2, 13, 30]. Various methods have been proposed to calculate chronological age through dental maturity and mineralization, among which we highlight those proposed by Nolla in 1960 [43] and by Demirjian et al. in 1973 [11].

The Demirjian method [11] is one of the most frequently used in estimating chronological age due to its simplicity, intra-examiner agreement, ease of standardization and ability to be reproduced, having been used and tested across a wide range of populations (e.g. [13, 15–17, 20, 29, 34, 38, 42, 50]. Various studies described in the literature have found the need for the method to be adapted to the populations in which it has been studied [36]. Rózelo-Kalinowska et al. [45] compared the dental age of 994 healthy Polish children from 6 to 16 years old with the development patterns proposed by Demirjian, finding that the method overestimated chronological age, with overestimation being more evident in 11-12-year-old girls and 13-year-old boys. Chen et al. [8] assessed the applicability of the method in 445 Chinese children between 8 and 16 years old, also finding overestimation of age. Qudeimat and Behbehani [44] tested the method with 509 healthy Kuwaiti children, finding they showed a delay in relation to the French-Canadian children used in the original study by Demirjian et al. [11]. Bagherpour et al. [1] tested the accuracy of the method with 170 girls and 114 boys in Iran, finding overestimation of age in boys of 0.34 years and 0.25 years in that of girls, despite concluding the method was appropriate for estimating age, especially in the 9 to 13-year-old sample. Then Jayaraman et al. [28] concluded that the method underestimated age by 0.24 years in a sample of 266 Chinese orthopantomographs. More recently, Sarkar et al. [46] analyzed the effectiveness of the method with a sample of 100 Indians between 5 and 24 years old, finding underestimation of age of 1.63 years in boys and 1.54 years in girls. Djukic et al. [13] assessed the accuracy of the Demirjian and Willems methods with 686 Serbian children between 4 and 15 years old, finding that both methods showed a discrepancy in relation to chronological age, although the Demirjian method was less accurate than that of Willems. Khorate et al. [30] found an underestimation of around 2 years of chronological age in a sample of 500 orthopantomographs of individuals between 4 and 22 years old in the State of Goa (India).

Besides the Demirjian method, this study analyzes the predictive capacities of the Nolla method [43] as it has shown itself to be very accurate with a sample of Spanish and Portuguese participants [48]. The Nolla [43] method has been one of the least frequently used and tested across populations, despite its effectiveness [4, 24, 47, 48]. This method has already been tested with children between 3 and 15 years old in Sweden [24], Finland [47], and Andalusia [4], giving a high correlation using a limited number of teeth (teeth 21, 46 and 43 for boys and 21, 47 and 46 for girls under 10 years of age). Holtgrave, Kretschmer, and Müller [27] also found high accuracy of the method, finding no significant differences between dental age and chronological age in girls (in boys the method overestimated chronological age). The study by Bolanos et al. [4] showed that the most reliable teeth for estimating chronological age through the Nolla method varied between boys and girls. Caro and Contreras [7] found greater reliability using this method in determining dental age in permanent teeth, in comparison to three other methods. With a sample of children of Bangladeshi and British Caucasian origin between 3 and 17 years of age, Maber et al. [38] found underestimation in all age groups, although more pronounced for girls and increasing particularly after 10 years old. Miloglu et al. [40] also found underestimation of chronological age in Turkish boys and girls from 6 to 18 years old, although more evident in girls, concluding that the method was only accurate for boys.

More recently, Kirzioglu and Ceyhan [31] compared the Nolla and Demirjian methods with a sample of 425 Turkish children between 7 and 13 years old, from the same socio-economic class and the same ethnic group. An underestimation of −0.53 years was found for boys and −0.57 for girls with the Nolla method, this method being more accurate between 9 and 11 years in both sexes and in the group of 13-year-old girls. On the other hand, the Demirjian method overestimated boys’ age by +0.52 and girls’ age by + 0.75. Kirzioglu and Ceyhan [31] concluded that these methods are not totally suitable, with it being necessary to assess specific tables for this population.

### Aims

The results of these previous studies indicate that estimating chronological age by means of dental mineralization stage is not a straightforward analysis. It therefore becomes essential to test the accuracy of estimating chronological age from age estimation methods and their applicability to different populations. In this article we intend to evaluate and compare the accuracy of estimating chronological age from dental age measured with the Demirjian and Nolla methods in a Portuguese and Spanish sample. The predicted accuracy of each method will be analyzed considering the global formula of prediction and taking the specific contribution of each tooth into account, according to the seven mandibular teeth staged according to Demirjian’s [11] and Nolla’s [43] dental maturity scales. In the prediction equations, consideration will also be given to variables such as sex and age-group (early childhood, late childhood, youth, and adulthood).

We propose the following specific objectives: 1) Analyze the predictive capacities of the methods proposed by Nolla [43] and Demirjian et al. [11]; 2) Compare the magnitude of agreement of the two methods; 3) construct a global model with a predictive value of chronological age according to the classifying variables that showed influence in determining the effectiveness of each method, namely sex and age-group (early childhood, late childhood, youth, and adulthood); 4) Carry out empirical testing of the model’s adjustment, comparing real values with predicted values; 5) Demonstrate that neither method has capacities to predict chronological age beyond youth and interpret reasons for the loss of predictive ability as age increases.

## Methods

### Participants

The sample comprises 821 orthopantomographs from Portuguese and Spanish Caucasian individuals, 409 male and 412 female, from Galicia (Spain) and the North of Portugal (convenience sample).The sample calculation was performed using the sample size determination formula based on the estimated proportion of the population with p (proportion of the population of individuals belonging to the category we are interested in studying) and q (proportion of the population of individuals not belonging to the category we are interested in studying, 1 – p) unknown (see [35]). So with a confidence level of 90% and α = 0.05, we would need at least 271 (rounded up from the figure of 270.6) subjects. Since we were studying elements of two populations (Portuguese and Spanish), although both Iberian, and since the Spanish population is more than double that of Portugal, we surveyed 270 Portuguese (32.89%) and 551 Spanish (67.11%) citizens. We previously checked there are no differences between Portuguese and Spanish populations. Indeed, the populations are very similar (both belong to Iberian Peninsula), and data were collected near the frontier between the two countries, for convenience reasons. So as expected, no differences were found and we decide to present the results together.

All participants are aged between 4 and 34 years, the mean (M) being 12.56 years and the standard-deviation (SD) 4.53 years. For males, ages range from 4.5 to 34 years, with M = 12.22 years and SD = 4.2 years. The age of females is between 4 and 28.6 years, with M = 12.91 years and SD = 4.8 years. The great majority are between 10 and 15 years of age (n = 334; 40.7%), followed by 7 to 10-year-olds (n = 204; 24.8%). We find a lower percentage of younger participants (3 to 7 years, n = 77; 9.4%) and older ones (20 to 35 years, n = 69; 8.4%) in the sample. We assessed teeth of the 3rd quadrant in all participants.

For age division of participants we used the criteria established in clinical pediatrics, according to Halcrow and Tayles [26], pp. 195–196], considering the childhood range as 1–15 years [19]: early childhood: 0–5 years; late childhood: 6–15 years. We considered as youth the ages from 15 to 19 years and adults beyond 19 years.

### Methods of age estimation

#### Demirjian method

The Demirjian method is a system based on eight stages (from A to H) of dental maturity in the seven left permanent mandibular teeth, observable through orthopantomographs. Each tooth was attributed a stage and converted in quantitative values by applying a specific table, the scores of the seven teeth are summed as a function of sex and the sum of dental maturity is obtained on a scale of 0 to100. This total is converted in dental age using a table for converting the results of dental maturity [11].

#### Nolla method

The Nolla method [43] allows classification of dental development from stage one (1 - no sign of calcification with the presence of crept), to stage ten (10 - apical end completed). The orthopantomograph of each tooth is assessed individually and compared with the stage of the Nolla table [43]. The dental age calculated corresponds to the sum of the Nolla scores. This method requires very consistent discrimination by the observer in assessing dental maturity through radiography [40].

### Ethics and procedures

The present study was part of research approved by the Faculty of Medicine at the University of Coimbra for Portuguese participants. All panoramic radiographs of Spanish participants are included in the database of personal information called “*File # 20: Patient management and clinical records of oral health*” (School of Medicine and Dentistry, University of Santiago de Compostela). Informed consent was obtained from all patients to include the orthopantomographs in this database, and their subsequent use for research purposes. For minors, the informed consent was requested from their parents/guardians.

The following radiological criteria of exclusion were applied: lack of clarity of dental structures due to problems of contrast, movement or artifacts; impacted teeth; radiopaque obturations or crowns; periapical lesions; endodontic treatment teeth; crowns bridging neighboring teeth.

### Statistical analysis

All statistical analyses were performed using the Statistical Package for Social Sciences (SPSS, version 22.0; SPSS Inc., Chicago, IL). Simple and multiple linear regressions were used to obtain a parsimonious model allowing estimation of chronological age from the measurements made of the seven teeth based on each method and the variables of sex and age-group. The statistical assumptions of the models were analyzed and fulfilled, namely those of normal distribution, homogeneity and independence of errors. The first two assumptions were validated graphically and the independence assumption was assessed with the Durbin-Watson statistic (values obtained close to 2) [39]. In the multiple regressions we used the VIF coefficients to diagnose multicollinearity and no variable showed VIF indicators of multicollinearity (all VIF < 10). For all analyses we considered a probability of type I error (α) = 0.05. We used cluster analysis to justify, and statistically demonstrate, the adequacy of age-groups formed.

### Inter-rater agreement and analysis of temporal consistency

Dental age was calculated for each method. In determining the inter-rater agreement, we calculated the Cohen’s kappa coefficient, for the set of seven teeth estimated by the Nolla method. To carry out the Cohen’s kappa, we made a partial examination of the codified data, asking four specialists to codify 72 individuals selected in a stratified and random way from those making up the sample. The kappa values for 7 separated teeth indicated an average Kappa of .79 for the Demirjian method and of .89 for the Nolla method. These values show there is clear convergence regarding the categorization decisions taken, since it is current practice to accept that values above 0.75 are indicators of good agreement [17]. We also obtained a good internal consistence for the teeth assessment, with α = 0.90 for the Demirjian method and α = 0.95 for the Nolla method (values above .80 are considered indicators of good reliability; [9]).

Aiming to analyze the temporal stability of the measurements made according to both methods, we calculated the Spearman correlation coefficient. Examination of the categorization made by the author of this study at two different times, separated by over six months, resulted in figures between 0.78 and 0.95 (all significant for *p <* .05), therefore showing good consistency over time. So we consider we can carry out the following analyses without subjectiveness in categorization being a reason for concern.

## Results

### Comparison between chronological and estimated age

All comparisons between chronological age and age estimated by the original Demirjian and Nolla methods, for males and females, showed significant differences (see Table 1). We found that for males, the Demirjian method overestimated chronological age, while the Nolla method underestimated it, with a statistically significant difference being recorded between the two methods. Comparisons for females point in the same direction, although the differences between the two methods and between these and chronological age were more pronounced. It is therefore important to elaborate regression equations adapted to the Iberian population (Portuguese and Spanish).

**Table 1:** **Comparisons between the chronological age and the estimated age by original Demirjian and Nolla methods, for males and females**

	Males				Females			
	Min	Max	M	SD	Min	Max	M	SD
**Age**								
Chronological (months)	54	203	132.62	35.55	42	203	132.10	35.70
Demirjian estimation	70	192	141.05	34.32	60	192	143.80	33.34
Nolla estimation	53	204	126.84	33.95	41	192	125.21	34.31
	**Difference between means**	**SE**	**Λ Wilks**	**F (2, 354)**	**Difference between means**	**SE**	**Λ Wilks**	**F (2, 354)**
**Comparisons**								
Chronological vs. Demirjian	−8.43***	0.80	0.40	261.18***	−11.70***	0.80	0.29	400.35***
Chonological vs. Nolla	5.78***	0.78			6.89***	0.81		
Demirjian vs. Nolla	14.21***	0.62			18.59***	0.66		

***p < .001.

### Chronological age forecast from the global scores of Demirjian and Nolla and tooth-by-tooth analysis

Analysis of the simple regression, when we took the sum of the seven teeth as the predictor and chronological age (measured in months) as dependent variable, was found to be statistically significant (see Table 2). We obtained a predictive capacity of 64.4% of total variance of chronological age of participants using the Nolla score and 47.5% using the Demirjian score (see for each method the range for unstandardized regression weights at 95% confidence interval). Setting out from the sample used and given the low rate of error associated with the inferential method used (under 1 possibility in 1000), the results showed we can apply the equations to Portuguese and Spanish individuals, in order to forecast their respective chronological ages, by both the Nolla and the Demirjian method. The formulas for forecasting chronological age using the Nolla and Demirjian scores are indicated in the last line of Table 2. Substituting the Demirjian or Nolla score in the equation by the value measured in each subject, we obtain an estimate of chronological age (in months). However, the predictive capacity was only slightly above 50% for the Nolla method and just under 50% for the Demirjian method. Confirmation of the predictive capacities of each tooth in particular may help us to clarify these results.

**Table 2:** **Simple regression analysis of chronological age predicted by the Nolla and Demirjian scores**

Model	Demirjian				Nolla			
	***B***	***SE B***	***β***	***t***	***B***	***SE B***	***β***	***t***
(Constant)	−168.01	11.80		−14.24***	−213.39	9.52		−22.42***
Predictor	3.45	0.13	.69	27.22***	5.78	0.15	.80	38.53***
R = .689, **R** ^**2**^ **= .475**, SEE = 3.28					R = .803**, R** ^**2**^ **= .644**, SEE = 2.70			
F(1,819) = 740.64, *p <* .001					F(1,819) = 1484.29, *p <* .001			
95% confidence interval for B: .267 to .308					95% confidence interval for B: .457 to .507			
**Equations forecasting chronological age through the significant teeth:**								
**Demirjian’s formula (global score):**					**Nolla’s formula (global score):**			
*Predicted chron. age* = −168.01 + 3.45 × Demirjian’s score					*Predicted chron. age* = −213.39+ 5.78 × Nolla’s score			

***p < .001.

Table 3 presents the result of the multiple regression analysis, taking as predictors the individual values of the seven teeth used in calculating the Demirjian and Nolla scores and maintaining the dependent variable of chronological age. We find that the seven teeth are able to predict around 57.7% with Demirjian scores and 74.5% with Nolla scores of total variance of chronological age (see for each method the range for unstandardized regression weights at 95% confidence interval). Introduction of the Demirjian or Nolla scores for each specific tooth as predictive variables in the regression equation allowed an increase in the predictive capacity of each method in relation to using the global score (rising from 47.5% to 57.7% in the Demirjian method and from 64.4% to 74.5% in the Nolla method).

**Table 3:** **Chronological age predicted by the teeth assessed by the Demirjian and Nolla scores**

Predictors:	Demirjian method				
	***B***	***SE B***	***β***	***t***	***95% CI for B***
(Constant)	−160.17	11.65		−13.75***	
Lateral Incisor	−5.24	1.48	-.18	−3.54***	0.04 to 0.41
Central Incisor	2.69	1.15	.11	2.33*	−0.68 to −0.19
Canine	4.02	1.96	.12	2.05*	0.01 to 0.66
1st Premolar	10.27	1.35	.36	7.59***	0.63 to 1.08
2nd Premolar	−0.35	1.83	-.01	−0.19	−0.33 to 0.27
1st Molar	0.26	0.76	.01	0.34	−0.10 to 0.15
2nd Molar	12.28	1.29	.46	9.52***	0.81 to 1.23
R_multiple_ = .759, **R** ^**2**^ **= .577**, SEE = 2.95, F(7,813) = 158.08, *p <* 0.001					
**Predictors:**	**Nolla method**				
(Constant)	67.29	19.72		3.41**	
Lateral Incisor	0.49	2.31	.01	0.21	−1.20 to - 0.18
Central Incisor	−8.27	3.12	-.09	−2.65**	−0.34 to 0.42
Canine	7.48	2.85	.16	2.62**	0.16 to 1.09
1st Premolar	−11.34	2.78	-.31	−4.08***	−1.40 to −0.49
2nd Premolar	15.32	2.50	.47	6.13***	0.87 to 1.69
1st Molar	−12.68	2.97	-.17	−4.27***	−1.54 to −0.57
2nd Molar	23.25	1.73	.72	13.47***	1.66 to 2.22
R_multiple_ = .863, **R** ^**2**^ **= .745**, SEE = 2.30, F(7,813) = 339.62, *p <* 0.001					
**Equations forecasting chronological age through the significant teeth:**					
**Demirjian’s formula (5 significant teeth):**			**Nolla’s formula (6 significant teeth):**		
*Predicted chron. age* = −158.77 + 2.68 × Central Incisor - 5.20 × Lateral Incisor + 3.95 × Canine + 10.22 × First Premolar + 12.25 × Second Molar			*Predicted chron. age* = 67.07 - 7.95 × Central Incisor + 7.57 × Canine − 11.29 × First Premolar + 15.29 × Second Premolar – 12.58 × First Molar + 23.24 × Second Molar		

*p < .05; **p < .01; ***p < .001.

We found that for the Demirjian method, the Second Premolar and the First Molar did not predict chronological age significantly (*p* > .05), while for the Nolla method only the Lateral Incisor was not significant. In both methods, the most significant predictor was the Second Molar; for the Demirjian method, the First Premolar was next, followed by the Lateral Incisor; for the Nolla method, the Second Premolar was next, followed by the First Molar and the Canine. Given the insignificance of the non-predictive teeth, we carried out new multiple regression equations excluding these teeth. The results of the equations forecasting chronological age through the significant teeth appear in the last line of Table 3. Substituting the respective Demirjian or Nolla scores in these equations, we obtain the predicted age, considering the sample of 4 to 34-year-olds.

### Influence of sex on predictive capacities of the Demirjian and Nolla methods

Since in previous studies (see Background to this article) both methods are shown to be sensitive to sex, we carried out regression analyses differentiated by sex. We took as predictor the global score of Demirjian and Nolla and as dependent variable the chronological age of men and women separately. The results are shown in Table 4.

**Table 4:** **Chronological age predicted by the Demirjian and Nolla methods for males and females**

Sex		Demirjian				Nolla			
		***B***	***SE B***	***β***	***t***	***B***	***SE B***	***β***	***t***
1) **Male**	Constant	-135.03	13.23		-10.20***	-186.28	11.14		-16.73***
	Predictor	3.10	0.15	.73	21.46***	5.36	0.18	.83	30.13***
R = .729, R^2^ = .531, SEE = 2.89						R = .831, R^2^ = .690, SEE = 2.35			
F(1,410) = 460.63, *p* < .001						F(1,407) = 907.54, *p* < .001			
95% confidence interval for B: .235 to .282						95% confidence interval for B: .418 to .476			
Demirjian’s formula (male): *Predicted chron. age* = -135.03 + 3.10 x Demirjian’s score						Nolla’s formula (male): *Predicted chron. age* = -186.28 + 5.36 x Nolla’s score			
2) **Female**	Constant	-225.68	21.25		-10.62***	-248.36	15.89		-15.63***
	Predictor	4.05	0.26	.67	18.01***	6.32	0.25	.78	25.53***
R = .665, R^2^ = .442,SEE = 3.58						R = .783, R^2^ = .614, SEE = 2.99			
F(1,410) = 324.50, *p* < .001						F(1,410) = 651.59, *p* < .001			
95% confidence interval for B: .301 to .374						95% confidence interval for B: .486 to .567			
Demirjian’s formula (female): *Predicted chron. age* = -225.68 + 4.05 x Demirjian’s score						Nolla’s formula (female): *Predicted chron. age* = -248.36 + 6.32 x Nolla’s score			

***p < .001.

The Demirjian method was found to predict 53.1% of total variance of chronological age for men and 44.2% for women, while the Nolla method predicted 69% of total variance for men and 61.4% for women. Therefore, and considering the magnitude of the regression coefficients (see Table 4), we found both methods are able to explain a greater proportion of total variance in men than in women. Comparison of the two methods indicated that the Nolla method had greater predictive capacity than the Demirjian method, for both men and women.

### Predictive capacities of the Demirjian and Nolla methods according to age-group

Aiming to sub-divide the sample in age groups as a function of subjects’ proximity in terms of accuracy of age estimation, we perform a Cluster Hierarchy Analysis (Ward method). For the Demirjian method, the dendrogram indicated the existence of two main clusters (cluster 1 – from 4 to 8 years, and cluster 2 – from 9 to 34 years), the second cluster being sub-divided in three clusters: from 9 to 14, from 15 to 18 years and from 19 to 34 years. For the Nolla method, the dendrogram presented an age-group distribution very close to that obtained for the Demirjian method: the Nolla method indicated the existence of two main clusters (cluster 1 – from 4 to 10 years, and cluster 2 – from 11 to 34 years), with the second cluster being sub-divided in another three: from 11 to 15 years, from 16 to 19 years and from 20 to 34 years. This procedure allowed us to respond to the objective of presenting the results differentiated according to age-groups, based on a similar distribution of explained variance from each method considering each age-group.

Table 5 presents the results of estimating chronological age for the four age-groups defined from the cluster analysis for the Demirjian and Nolla methods. Each cluster had a similar configuration in terms of estimation of chronological age from the Demirjian and Nolla scores.

**Table 5:** **Chronological age predicted by the Demirjian and Nolla methods for four age-groups defined by cluster analysis**

Age-group		Demirjian				Age-group	Nolla			
		***B***	***SE B***	***β***	***t***		***B***	***SE B***	***β***	***t***
**4 to 8 years**	Constant	67.26	1.65		40.84***	**4 to 10 years**	54.85	1.69		32.44***
	Predictor	0.20	0.02	.69	11.99		0.43	0.02	.85	28.43***
R = .688, R^2^ = .473, SEE = 0.69						R = .846, R^2^ = .715, SEE = 0.80				
F(1,410) = 143.71, *p* < .001						F(1,322) = 808.14, *p* < .001				
95% confidence interval for B: .047 to 0.65						95% confidence interval for B: .191 to .220				
*Predicted chron. age* = 67.26 + 0.20 x Demirjian’s score						*Predicted chron. age* = 54.85 + 0.43 x Nolla’s score				
**9 to 14 years**	Constant	4.02	7.855		0.51	**11 to 15 years**	53.15	6.75		7.88***
	Predictor	0.83	.049	.64	17.07***		0.59	0.04	.65	15.22***
R = .640, R^2^ = .410, SEE = 1.29						R = .654, R^2^ = .428, SEE = 1.00				
F(1,410) = 291.46, *p* < .001						F(1,310) = 231.75, *p* < .001				
95% confidence interval for B: .208 to .262						95% confidence interval for B: .248 to .322				
*Predicted chron. age* = 4.02 + 0.83 x Demirjian’s score						*Predicted chron. age* = 53.15 + 0.59 x Nolla’s score				
**15 to 18 years**	Constant	31.17	40.94		0.76	**16 to 19 years**	59.68	59.44		1.00
	Predictor	0.93	.234	.32	3.94***		0.78	0.32	.24	2.46*
R = .324, R^2^ = .105, SEE = 1.08						R = .237, R^2^ = .056, SEE = 1.20				
F(1,410) = 15.49, *p* < .001						F(1,102) = 6.05, *p* = .016				
95% confidence interval for B: .129 to .390						95% confidence interval for B: .072 to .675				
*Predicted chron. age* = 31.17 + 0.93 x Demirjian’s score						*Predicted chron. age* = 59.68 + 0.78 x Nolla’s score				
**19 to 34 years**	Constant	-122.97	809.89		-0.15	**20 to 34 years**	-214.81	362.66		-0.59
	Predictor	2.14	4.58	.05	0.47		2.50	1.90	.147	1.33
R = .047, R^2^ = .002, SEE = 2.28						R = .147, R^2^ = .022, SEE = 2.16				
F(1,410) = 0.22, *p* = .641						F(1,79) = 1.74, *p* = .191				
95% confidence interval for B: -1.96 to 3.17						95% confidence interval for B: -.615 to 3.03				
(Demirjian method not statistically significant, *p* > .05)						(Nolla method not statistically significant, *p* > .05)				

*p < .05; ***p < .001.

As observed in Table 4, as the age-group rises, the predictive capacity falls, for both the Demirjian and the Nolla methods. Both methods showed greater predictive power in the youngest age-group (4 to 8 years for the Demirjian method and 4 to 10 years for the Nolla method) and neither was significant in adults (19 to 34 years for the Demirjian method and 20 to 34 years for the Nolla method).

**Figure 1:**
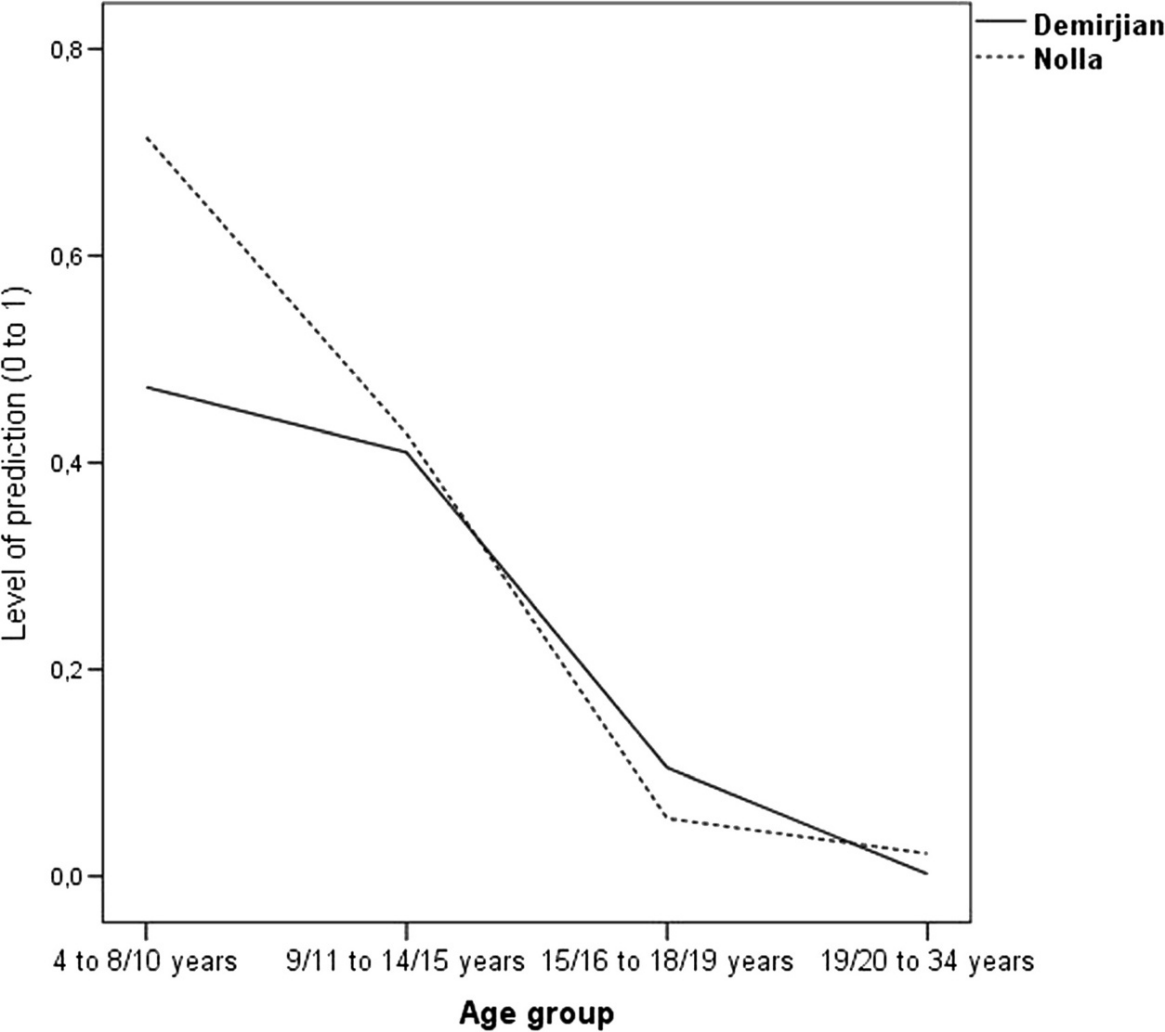
**Level of prediction of the Demirjian and Nolla methods by age group.**

We also found forecasting differences between the two methods, Nolla being more accurate than Demirjian for the youngest ages (early and late childhood). Using the Nolla method, a forecasting gain of 24.2% (R^2^_Nolla_ = .715 - R^2^_Demirjian_ = .473) is obtained over the Demirjan method, for children in the first age group (4–8 years for the Demirjian method and 4–10 years for the Nolla method). As for the second age group (9–14 years for the Demirjian method and 11–15 years for the Nolla method), the forecasting gain using the Nolla method was very small, showing better forecasting of only 1.84% (R^2^_Nolla_ = .428 - R^2^_Demirjian_ = .410). As for the third age group (15–18 years for the Demirjian method and 16–19 years for the Nolla method), although both methods lost considerable predictive capacity in relation to the previous age-groups, the forecasting gains were greater using the Demirjian method, with a 4.9% higher forecast (R^2^_Demirjian_ = .105 - R^2^_Nolla_ = .056) compared to the Nolla method. Figure 1 illustrates the predictive capacities of each method as a function of participants’ age-group.

### Predictive capacities of the Demirjian and Nolla methods for the three significant age groups according to sex

From the results presented in the previous section, it is important to analyze in more detail the predictive capacities of both methods up to the age where the method still showed predictive capacities (18 years in the Demirjian method and 19 in the Nolla method). Tables 6 and 7 present the regression analysis of both methods for the three significant age-groups, for males and females.

**Table 6:** **Chronological age (years) predicted by the Demirjian and Nolla methods for three age-groups for males**

Age-group		Demirjian				Age-group	Nolla			
		***B***	***SE B***	***β***	***t***		***B***	***SE B***	***β***	***t***
**4 to 8 years**	Constant	2.87	0.43		6.66***	**4 to 10 years**	-3.11	0.56		-5.52***
	Predictor	0.06	0.01	.73	10.09***		0.21	0.01	.85	20.29***
R = .732, R^2^ = .537, SEE = 0.66						R = .849, R^2^ = .721, SEE = 0.77				
F(1,88) = 101.87, *p* < .001						F(1,159) = 411.73, *p* < .001				
95% confidence interval for B: .047 to .070						95% confidence interval for B: .190 to .231				
*Predicted chron. age* = 2.87 + 0. 60 x Demirjian’s score						*Predicted chron.* age = -3.11+ 0.21 x Nolla’s score				
**9 to 14 years**	Constant	-9.21	1.55		-5.93***	**11 to 15 years**	-4.54	1.38		-3.28**
	Predictor	0.22	0.02	.68	13.43***		0.27	0.02	.70	12.69***
R = .679, R^2^ = .461, SEE = 1.23						R = .698, R^2^ = .487, SEE = 0.93				
F(1,211) = 180.40, *p* < .001						F(1,170) = 161.11, *p* < .001				
95% confidence interval for B: .189 to .254						95% confidence interval for B: .224 to .306				
*Predicted chron. age* = -9.21 + 0.22 x Demirjian’s score						*Predicted chron.* age = -4.54 + 0.27 x Nolla’s score				
**15 to 18 years**	Constant	-5.68	7.14		-0.80	**16 to 19 years**	-12.89	14.68		-0.88
	Predictor	0.22	0.07	.35	3.03**		0.43	0.21	.29	2.04*
R = .345, R^2^ = .119, SEE = 1.13						R = .289, R^2^ = .083, SEE = 1.16				
F(1,68) = 9.19, *p* = .003						F(1,46) = 4.18, *p* = .047				
95% confidence interval for B: .074 to .361						95% confidence interval for B: .007 to .857				
*Predicted chron. age* = -5.68 + 0.22 x Demirjian’s score						*Predicted chron. age* = -12.89+ 0.43 x Nolla’s score				

**p < .01; ***p < .001.

**Table 7:** **Chronological age (years) predicted by the Demirjian and Nolla methods for three age-groups for females**

Age-group		Demirjian				Age-group	Nolla			
		***B***	***SE B***	***β***	***t***		***B***	***SE B***	***β***	***t***
**4 to 8 years**	Constant	2.23	0.52		4.31***	**4 to 10 years**	-3.49	0.58		-5.99***
	Predictor	0.06	0.01	.74	9.33***		0.21	0.01	.85	20.66***
R = .742, R^2^ = .551, SEE = 0.67						R = .852, R^2^ = .726, SEE = 0.81				
F(1,71) = 87.05, *p* < .001						F(1,161) = 417.78, *p* < .001				
95% confidence interval for B: .045 to .074						95% confidence interval for B: .191 to .232				
*Predicted chron. age* = 2.23 + 0. 06 x Demirjian’s score						*Predicted chron.* age = -3.49 + 0.21 x Nolla’s score				
**9 to 14 years**	Constant	-20.66	2.17		-9.51***	**11 to 15 years**	-14.18	2.42		-5.85***
	Predictor	0.33	0.02	.72	14.72***		0.40	0.04	.69	11.18***
R = .715, R^2^ = .511, SEE = 1.17						R = .689, R^2^ = .475, SEE = 1.00				
F(1,207) = 216.71, *p*< .001						F(1,138) = 124.95, *p* < .001				
95% confidence interval for B: .288 to .376						95% confidence interval for B: .330 to .472				
*Predicted chron. age* = -20.66 + 0.33 x Demirjian’s score						*Predicted chron.* age = -14.18 + 0.40 x Nolla’s score				
**15 to 18 years**	Constant	-103.39	32.59		-3.17**	**16 to 19 years**	-4.85	15.33		-0.32
	Predictor	1.20	0.33	.42	3.66**		0.32	0.22	0.192	1.44
R = .422, R^2^ = .178, SEE = 0.97						R = .192, R^2^ = .037, SEE = 1.25				
F(1,62) = 13.44, *p* = .001						F(1,54) = 2.07, *p* = .156				
95% confidence interval for B: .608 to 1.79						95% confidence interval for B: -.125 to .759				
*Predicted chron. age* = -103.39 + 1.20 x Demirjian’s score						(Nolla method not statistically significant, *p* > .05)				

**p < .01; ***p < .001.

We found that for males up to 15 years of age, the Nolla method was a more significant predictor. More specifically, the Nolla method was shown to predict 72.1% of chronological age of boys up to 10 years old, while the Demirjian method only had predictive capacity of 53.7% (up to 8 years old). For the second age-group, the methods were close in terms of predictive power: Nolla presented a predictive capacity of 48.7% of the chronological age of late childhood between 11 and 15, while Demirjian predicted around 46.1%. Surprisingly, the Nolla method’s tendency to possess greater predictive power than the Demirjian method was inverted for young boys: the Demirjian method showed a predictive capacity of 11.9% of the chronological age of boys between 15 and 18, whereas the Nolla method only managed to predict 8.3% of the chronological age of boys from 16 to 19. Our results point to the Nolla method being a better predictor of the chronological age of boys up to late childhood, while for teenagers, despite both methods losing considerable predictivepower, the Dermirjian method is seen to be more effective.

Considering females, we found similar results to males only in childhood, although for girls both methods were seen to be slightly more accurate. More specifically, up to 10 years of age, the Nolla method was shown to be a more effective predictor of the chronological age of girls (72.6% of predictive capacity), since the Demirjian method revealed a lower predictive power (around 55.1% up to 8 years old). From late childhood, this tendency was inverted, with the Demirjian method showing itself to be a more accurate predictor: the Demirjian method presented a predictive capacity of 51.1% of the chronological age of girls between 9 and 14 and the Nolla method predicted around 47.5% of the chronological age of girls between 11 and 15. In youths, the differences between the methods were even more marked, with the Nolla method not showing predictive power in females from 16 to 19. On the contrary, the Demirjian method was a significant predictor of females from 15 to 18, explaining about 17.8% of their chronological age. Similarly to the results obtained for males, in females, the Nolla method’s tendency to be a more significant predictor in girls up to late childhood was confirmed. Beyond this age the tendency was inverted, the Demirjian method showing itself to be a better predictor and managing to be significant up to 18 years of age, while Nolla was only able to predict the chronological age of girls up to 15.

## Discussion

Far from presenting consensual results, the research carried out led us to the need to test the applicability and credibility of the Nolla and Demirjian methods with a sample of the Portuguese and Spanish population. Substituting in the corresponding equation the Nolla [43] or Demirjan et al. [11] value, the resulting forecast is accurate, forming a useful instrument for diagnosis and treatment planning. The seven teeth considered by the Demirjian method were able to predict 47.5% of the chronological age of participants and the Nolla method predicted 64.4%. In our sample, therefore, the Nolla method was a more significant predictor.

The accuracy of both methods varied between the sexes and age groups. Both methods were found to be more precise with males. Estimation of chronological age for four age-groups defined from the cluster analysis showed that as the age-group increases, the predictive capacities of both methods diminish. The Nolla method was more accurate than the Demirjian method in early and late childhood for both sexes. Neither method could predict chronological age in adults. For girls, the Nolla method was more significant up to late childhood and the Demirjian method from this age up to 18 years old. Since the sex variable influenced the predictive capacities of both methods, we conclude that equations to estimate chronological age should use specific coefficients for males and females. Indeed, it is a fact that teeth appear in females before males [13, 25]. The dental maturity process also presents sex differences, and mineralization is also earlier in females than in males [13, 25, 37]. The earliest phases of dental maturity are very similar in both sexes up to the first onset of menstruation, greater development occurring beyond that period in girls [3].

We found the Demirjian method tends to overestimate the real age of participants and the Nolla method tends to underestimate it. Using Demirjian led systematically to overestimation of chronological age. Among others, overestimation was found in the studies by Frucht et al. [21], Willems et al. [50], Eid et al. [14], Foti et al. [20], Maber [38], Chen et al. [8], Galic et al. [22], Lee et al. [33], Nik-Hussein et al. [42], Lee et al. [34], Feijóo et al. [15, 16], Kirzioglu and Ceyhan [31], Flood et al. [18], Jayaraman et al. [29] and Djukic et al. [13]. Among the studies finding overestimation, we should mention those of Khorate et al. [30] with individuals in Goa, India, Sarkar et al. [46] with Indian individuals, Cruz-Ladeira et al. [10] with Venezuelan individuals and Chen et al. [8] with Chinese children. More recently, Jayaraman et al. [29] performed a meta-analysis of 274 studies where the Demirjian method was used. The authors recorded average overestimation of chronological age in all the studies (of 0.60 years for boys and 0.65 years for girls), except in a Chinese sample of boys and a Venezuelan one with boys and girls.

Few studies have investigated use of the Nolla method in different populations. One of the most recent studies was made by Kirzioglu and Ceyhan in 2012 [31]. The authors compared the methods of Nolla [43], Havikko [24] and Demirjian et al. [11] with a sample of healthy Turkish children between 7 and 13 years old, from the same socio-economic status and the same ethnic group. Underestimation of dental age was found with the Nolla method, being more accurate for chronological age between 9 and 11 years in both sexes and in the group of 13-year-old girls. Kirzioglu and Ceyhan [31] concluded that, despite the Nolla method being more accurate than those of Havikko and Demirjian, these three methods are not completely adjusted, it being necessary to define specific tables for that population. Bolanos et al. [4] used the Nolla method with a Spanish sample, finding high accuracy for boys and girls up to 10 years old, results which are in agreement with ours. Caro and Contreras [7] also found higher accuracy in the Nolla method than in the other methods they tested.

One of the possible explanations we point out for the Nolla method showing itself to be a more significant predictor may be related to the fact this method presents more inter-stage sub-divisions, allowing greater inter-stage differentiation of dental maturity. Another explanation could be because in the Demirjian method, the Second Premolar and the First Premolar were not significant in predicting participants’ chronological age and the Canine was a weak predictor. However, for the Nolla method, only the Lateral Incisor was not significant in predicting chronological age, all the other teeth being good predictors.

The exclusion of insignificant teeth increased the predictive capacity of the Demirjian method to 57.7% and that of Nolla to 74.5%. The better predictive capacities of Nolla compared to Demirjian is therefore demonstrated in a more heterogeneous sample regarding age (4–35 years), whether using the Nolla value [43] or the Demirjian et al. [11] global one, or when considering the specific influence of each tooth. Our results are in line with studies indicating the existence of inaccuracies in estimating chronological age, above all in the Demirjian method – Frucht et al. [21], Willems et al. [50], Eid et al. [14], Foti et al. [20], Maber [38], Chen et al. [8], Liversidge [36], Cruz-Ladeira et al. [10], Galic et al. [22], Lee et al. [33], Nik-Hussein et al. [42], Feijóo et al. [15, 16], Kirzioglu and Ceyhan [31], Flood et al. [18], Djukic et al. [13], Sarkar et al. [46], and Khorate et al. [30].

In all the studies which found significant differences between chronological age and dental age using the various methods, the authors have suggested using correction factors. According to Nik-Hussein et al. [42], the discrepancies between chronological age and dental age can be due to a positive secular general tendency in which growth and somatic development could contribute to earlier dental eruption and mineralization.

Our results agree with previous studies carried out in a variety of geographical locations, ethnic groups, nutritional and socio-economic conditions, which arrived at different results in relation to the original populations that gave rise to the methods, with the need to elaborate forecasting equations appropriate for each population [1, 32, 47].

## Conclusions

We can estimate accurately the chronological age for early and late childhood using the Nolla and Demirjian et al. methods. Including adults leads to reducing the predictive capacities of both methods, and especially that of Demirjian.

The Nolla method showed greater predictive capacities than the Demirjian one in samples with more heterogeneous ages. The predictive capacity of both methods was significantly higher for boys and girls up to 10 years old, diminishing progressively up to 18 years old and ceasing to be significant beyond that age. Of all the age segments, the most favorable with the Demirjian method was up to 8 years old, and with the Nolla method it was up to 10 years, for both sexes. The Demirjian method tends to overestimate chronological age and the Nolla method tended to underestimate it, although the differences were less in relation to real age. It is therefore important to elaborate regression equations adapted to the populations studied.

Participants’ sex affected the magnitude of predictive capacities in both methods. The Nolla method was more accurate than the Demirjian method in early and late childhood for both sexes. For girls, the Nolla method was more significant up to late childhood and the Demirjian method from this age up to 18 years old compared to that of Nolla. It is therefore important to elaborate regression equations adapted to the populations studied, differentiating by sex and age-group.

## Abbreviations

B: Unstandardized regression coefficient
chron. age: Chronological age
Dem.: Demirjian method
F: F-test
ICC: Intra-class correlation coefficient
M: Mean
Max: Maximum value
Min: Minimum value
*P*: Significance level
R: Linear regression coefficient
R^2^: R squared (coefficient of determination)
SD: Standard-deviation
SE B: Standard-error for unstandardized coefficient
SE: Standard-error
SEE: Standard-error of the estimate
T: Student’s t-test
Β: Standardized coefficient
Λ: Wilks’ Lambda.

## References

[1] Bagherpour A, Imanimoghaddam M, Bagherpour MR, Einolghozati M (2010). Dental age assessment among Iranian children aged 6–13 years using the Demirjian method. Forensic Sci Int.

[2] Birch W, Dean MC (2014). A method of calculating human deciduous formation times and of estimating the chronological ages of stressful events occurring during deciduous enamel formation. J Forensic Leg Med.

[3] Blenkin M, Taylor J (2012). Age estimation charts for a modern Australian population. Forensic Sci Int.

[4] Bolaños MV, Manrique MV, Bolaños MJ, Briones MT (2000). Approaches to chronological age assessment based on dental calcification. Forensic Sci Int.

[5] Camerieri R, Ferrante L, Liversidge HM, Prieto JL, Brkic H (2008). Accuracy of age estimation in children using radiograph of developing teeth. Forensic Sci Int.

[6] Cardoso HF (2007). Accuracy of developing tooth length as an estimate of age in human skeletal remains: The deciduous dentition. Forensic Sci Int.

[7] Caro AC, Contreras IC (2001). Análisis y comparación de cuatro métodos radiográficos para determinar la edad dental (maduración dental) en dientes permanentes. Int J Dental Anthropol.

[8] Chen JW, Guo J, Zhou J, Liu RK, Chen TT, Zou SJ (2010). Assessment of dental maturity of western Chinese children using Dermijian’s method. Forensic Sci Int.

[9] Cohen J (1988). Statistical Power Analysis for the Behavioural Sciences.

[10] Cruz-Ladeira A, Linares-Argote J, Martinez-Rodriguez M, Rodriguez-Calvo MS, Otero XL, Concheiro L (2010). Dental age estimation in Spanish and Venezuelan children. Comparison of Demirjian and Chaillet’s scores. Int J Legal Med.

[11] Demirijian A, Goldstein H, Tanner JM (1973). A new system of dental age assessment. Hum Biol.

[12] Diz P, Limeres J, Salgado AFP, Tomás I, Delgado LF, Vázquez E, Feijoo JF (2011). Correlation between dental maturation and chronological age in patients with cerebral palsy, mental retardation, and Down syndrome. Res Dev Disabil.

[13] Djukic K, Zelic K, Milenkovic P, Nedeljovic N, Djuric M (2013). Dental age assessment validity of radiographic methods on Serbian children population. Forensic Sci Int.

[14] Eid R, Simi R, Friggi M, Fisberg M (2002). Assessment of dental maturity of Brazilian children aged 6 to 14 years using Demirjian’s method. Int J Paediatric Dent.

[15] Feijoo G, Barberia A, De Nova J, Prieto JL (2012). Dental age estimation in Spanish children. Forensic Sci Int.

[16] Feijoo G, Barberia A, De Nova J, Prieto JL (2012). Permanent teeth development in a Spanish sample. Application to dental age estimation. Forensic Sci Int.

[17] Fleiss J (2003). Statistical Methods for Rates and Proportions.

[18] Flood SJ, Franklin D, Turlach BA, McGeachie J (2013). A comparison of Demirjian’s four dental development methods for forensic age estimation in South Australian sub-adults. J Forensic Leg Med.

[19] Forfar JO, Arneil GC, Campbell AGM, McIntosh N (1998). Forfar and Arneil’stextbook of Paediatrics.

[20] Foti B, Llys L, Adalian P, Giustiniani J, Maczel M, Signoli M, Dutour O, Leonetti G (2003). New forensic approach to age determination in children based on tooth eruption. J Forensic Sci Int.

[21] Frucht S, Schnegelsberg C, Schulte-Mönting J, Rose E, Jonas I (2000). Dental age in southwest Germany. A radiographic study. J Orofac Orthop.

[22] Galic I, Nakas E, Prohic S, Selimovic E, Obradovic B, Petrovecki M (2010). Dental age estimation among children aged 5–14 years using the Demirjian method in Bosnia-Herzegovina. Acta Stomatol Croat.

[23] Garn SM, Lewis AB, Kerewsky RS (1965). Genetic, nutritional, and maturational correlates of dental development. J Dent Res.

[24] Haavikko K (1974). Tooth formation age estimated on a few selected teeth: a simple method for clinical use. Proc Finn Dent Soc.

[25] Hagg U, Taranger J (1981). Dental emergence stages and the pubertal growth spurt. Acta Odontol Scand.

[26] Halcrow SE, Tayles N (2008). The bioarchaeological investigation of childhood and social age: problems and propects. J Archaeol Method Theory.

[27] Holtgrave EA, Kretschmer R, Müller R (1997). Acceleration in dental development: fact or fiction. Eur J Orthod.

[28] Jayaraman J, Roberts GJ, King NM, Wong HM (2012). Dental age assessment of southern Chinese using the United Kingdom Caucasian reference dataset. Forensic Sci Int.

[29] Jayaraman J, Wong HM, King NM, Roberts GJ (2013). The French-Canadian data set of Demirjian for dental age estimation: a systematic review and meta-analysis. JForensic Leg Med.

[30] Khorate MM, Dinkar AD, Ahmed J (2014). Accuracy of age estimation methods from orthopantomograph in forensic odontology: a comparative study. Forensic Sci Int.

[31] Kirzioglu Z, Ceyhan D (2012). Accuracy of different dental age estimation methods on Turkish children. Forensic Sci Int.

[32] Koshy S, Tandon S (1998). Dental age assessment: the applicability of the Demirjian’s method in south Indian children. Forensic Sci Int.

[33] Lee SS, Byuin YS, Park MJ, Choi JH, Yoon CL, Shin KJ (2010). The chronology of second and third molar mineralization in Koreans and its application to forensic age estimation. Int J Legal Med.

[34] Lee SS, Kim D, Lee S, Lee UY, Seo JS (2011). Validity of Demirjian’s and modified Demirjian’s methods in age estimation for Korean juveniles and adolescents. Forensic Sci Int.

[35] Levine DM, Berenson ML, Stephan D (2014). Estatística: Teoria e aplicações usando Microsoft Excel em Português.

[36] Liversidge HM (2010). Interpreting group differences using Demirjian’s dental maturity method. Forensic Sci Int.

[37] Luca SD, Giorgio SD, Buttu AC, Biagi R, Cingolani M, Cameriere R (2012). Age estimation in children by measurement of open apices in tooth roots: Study of a Mexican sample. Forensic Sci Int.

[38] Maber M, Liversidge HM, Hector MP (2006). Accuracy of age estimation of radiographic methods using developing teeth. Forensic Sci Int.

[39] Marôco J (2011). Análise Estatística com o SPSS Statistics.

[40] Miloglu O, Celikoglu M, Dane A, Cantekin K, Yilmaz AB (2011). Is the assessment of dental age by the Nolla method valid for Eastern Turkish children?. J Forensic Sci.

[41] Moradi M, Sirous M, Morovatti P (2012). The reliability of skeletal age determination in an Iranian sample using Grelich and Pyle method. Forensic Sci Int.

[42] Nik-Hussein NN, Kee KM, Gan P (2011). Validity of Demirjian and Willems methods for dental age estimation for Malaysian children aged 5–15 years old. Forensic Sci Int.

[43] Nolla CM (1960). The development of the permanent teeth. J Dent Child.

[44] Qudeimat MA, Behbehani F (2009). Dental age assessment for Kuwait children using Demirjian’s method. Ann Hum Biol.

[45] Rózylo-Kalinowska I, Kiworkowa-Raczkowska E, Kalinowski P (2008). Dental age in Central Poland. Forensic Sci Int.

[46] Sarkar SS, Kailasam SB, Kumar PM (2013). Accuracy of estimation of dental age in comparison with chronological age in Indian population: a comparative analysis of two formulas. J Forensic Leg Med.

[47] Staaf V, Mörnstad H, Welander U (1991). Age estimation based on tooth development: a test of reliability and validity. Scand J Dent Res.

[48] Tomás LF, Tomás I, Varela-Patiño P, Salgado AFP, Mónico LS, Martin-Biedma B (2014). Dental age estimation with Nolla method in a Spanish and Portuguese sample. Sylwan.

[49] Tunc ES, Koyuturk AE (2008). Dental age assessment using Demirjian method on northern Turkish children. Forensic Sci Int.

[50] Willems VG, Van-Olmen A, Spiessens B, Carels C (2001). Dental age estimation in Belgian children: Demirjian’s technique revisited. J Forensic Sci Int.

[CR51] The pre-publication history for this paper can be accessed here: http://www.biomedcentral.com/1472-6831/14/160/prepub

